# Adoptive Cell Therapy in Hepatocellular Carcinoma: Biological Rationale and First Results in Early Phase Clinical Trials

**DOI:** 10.3390/cancers13020271

**Published:** 2021-01-13

**Authors:** Philippe Rochigneux, Brice Chanez, Bernadette De Rauglaudre, Emmanuel Mitry, Christian Chabannon, Marine Gilabert

**Affiliations:** 1 Department of Medical Oncology, Paoli-Calmettes Institute, 232 Boulevard Sainte Marguerite, 13009 Marseille, France; chanezb@ipc.unicancer.fr (B.C.); DERAUGLAUDREB@ipc.unicancer.fr (B.D.R.); MITRYJE@ipc.unicancer.fr (E.M.); GILABERTM@ipc.unicancer.fr (M.G.); 2 Team Immunity and Cancer, Centre de Recherche en Cancérologie de Marseille (CRCM), INSERM U1068, CNRS UMR 7258, Paoli-Calmettes Institute, Aix-Marseille University, 13009 Marseille, France; 3 Centre for Clinical Investigation in Biotherapy, Paoli-Calmettes Institute, Aix-Marseille University, INSERM CBT 1409, 13009 Marseille, France; CHABANNONC@ipc.unicancer.fr

**Keywords:** hepatocellular carcinoma, immunotherapy, adoptive cell transfer, CAR-T cells, engineered TCR

## Abstract

**Simple Summary:**

The mortality of hepatocellular carcinoma (HCC) is quickly increasing worldwide. Immunotherapy enables the immune defense of the organism to target liver cancer cells. Recent technologies enable engineering of immune cells, and notably T lymphocytes, to make them more efficient against the tumor. These technics (called TCR engineered T cells and CAR-T cells) are promising and are actually tested in clinical trials. This review explains the concept of TCR modified and CAR-T cells in liver cancer (targets and mechanisms of action) and reports the results from recent clinical trials.

**Abstract:**

The mortality of hepatocellular carcinoma (HCC) is quickly increasing worldwide. In unresectable HCC, the cornerstone of systemic treatments is switching from tyrosine kinase inhibitors to immune checkpoints inhibitors (ICI). Next to ICI, adoptive cell transfer represents another promising field of immunotherapy. Targeting tumor associated antigens such as alpha-fetoprotein (AFP), glypican-3 (GPC3), or New York esophageal squamous cell carcinoma-1 (NY-ESO-1), T cell receptor (TCR) engineered T cells and chimeric antigen receptors (CAR) engineered T cells are emerging as potentially effective therapies, with objective responses reported in early phase trials. In this review, we address the biological rationale of TCR/CAR engineered T cells in advanced HCC, their mechanisms of action, and results from recent clinical trials.

## 1. Introduction

Primary liver cancer, mainly hepatocellular carcinoma (HCC), is the second leading cause of cancer deaths worldwide [1]. The incidence of HCC is increasing from 14 million cases worldwide in 2012 to an expected 22 million cases in 2030 [2], mainly due to the increasing incidence of chronic alcohol use and nonalcoholic steatohepatitis (NASH) in Western countries [3] and the endemicity of viral hepatitis in African and Asian countries leading to cirrhosis [4]. Liver transplantation is currently the optimal definitive treatment for HCC [5,6] but is reserved for early stage cancers. If tumor resection, transarterial chemotherapies, and percutaneous therapies are more commonly used in practice to cure localized HCC, advanced HCC still has a fatal prognosis [7]. Systemic therapy is a key treatment option in patients with portal vein involvement, extra-hepatic extension, disease recurrence after surgery and in progressors after liver-directed therapy. Various systemic therapies, including cytotoxic chemotherapy (doxorubicin), interferon-alpha, and hormonotherapy (tamoxifen, megestrol anti-androgens) have been tested with very limited clinical benefits and significant toxicities [8]. Indeed, HCC is classically considered as a chemoresistant disease as the median overall survival (OS) of advanced HCC treated with doxorubicin is 10.6 weeks [9]. In 2008, the SHARP trial showed encouraging results of sorafenib, an oral tyrosine kinase inhibitor (TKI), with a median OS of 10.7 months vs. 7.9 months as compared to placebo; consequently sorafenib became the standard treatment in first line advanced HCC [10]. Then there was a sad saga of a decade where no systemic therapeutic agent showed OS benefits. However, other TKIs such as regorafenib [11], cabozantinib [12], lenvatinib [13], and a vascular endothelial growth factor receptor 2 (VEGFR2)—such as the monoclonal antibody ramucirumab [14]—demonstrated improved outcomes compared to placebo in first and second line of treatment.

Though immunotherapy with cytokines (interferon alpha-2b, interleukin-12) did not yield in encouraging result, randomized controlled trial reported the benefit of immune checkpoint inhibitors (ICI) in advanced HCC. In second line, nivolumab (CheckMate 040) and pembrolizumab (Keynote 240) showed signs of activity (response rate of 14% and 17%, respectively) but without major clinical benefit [15,16]. Published in 2020, the phase III combination atezolizumab plus bevacizumab in first line demonstrated a superiority in OS compared to sorafenib (HR = 0.58, 95 CI 0.42–0.79, *p* < 0.001), leading to the FDA approval of the association [17]. Despite this progress, the clinical outcomes in advanced HCC remain very poor with an OS at 12 months of 67.2% with atezolimumab-bevacizumab. As first evidences of immunotherapy are emerging, there is a need for additional immuno-oncological options [18,19].

The liver is an organ with a very specific immune system [20]. First, HCC is considered as an immunogenic tumor because of his anatomic position allowing the detection of pathogens entering by the gut, processing by many phagocytic cells (e.g., Kupffer cells) and innate immune cells (e.g., NKT and iNKT cells). Besides, the liver also has multiple subtypes of CD4^+^ T cells with immunomodulatory functions and cytotoxic CD8^+^ T cells. However, even if these memory cells can help eradicating the tumor [21], they are rarely able to control advanced HCC by themselves. Second, the cirrhosis around HCC cells is also an unique background. The liver continuously removes a large spectrum of pathogens from the circulation while ensuring organ protection by maintaining immunotolerance [22]. However, in chronic liver disease (necroinflammation), proinflammatory signals (IL-2, IL-7, IL-12, IL-15, and IFN-γ) break this tolerance leading to continuous cell death, compensatory regeneration, and liver fibrosis, which collectively induce tumorigenesis. The immune system is also dysregulated due to anti-inflammatory cytokines (IL-10, IL-13, and TGF-β) leading to the suppression of effective anti-tumor immune responses [22].

Consequently, driven by the success observed in hematology, researchers engineered cytotoxic cells (mainly CD8^+^ and rarely NK cells) targeting HCC to increase their cytotoxic properties [23]. Up to date, adoptive cells transfer (ACT) success in solid tumors was exceptional [24,25]. Due to the presence of tumor associated antigens (TAA) with an acceptable specificity, HCC in one of the most promising organ for ACT in solid tumors [26]. In this report, we will review the biological rationale of adoptive cell transfer in advanced HCC, the results of ACT published clinical trials and the setting of the ongoing trials. Finally, we will discuss the main concerns and perspectives of this emerging field.

## 2. Biological Rationale of Adoptive Cell Transfer in Hepatocellular Carcinoma 

### 2.1. Concept of CAR/TCR Engineered T Cells

After decades of relatively low success rates when trying to convert immunological concepts in efficacious immunotherapeutic tools—with the possible exception of allogeneic hematopoietic cell transplantation that was empirically developed as a cellular immunotherapy to treat mostly hematological malignancies—recent years have witnessed the introduction of several practices changing medicinal products. In particular, the remarkable success rates and improvement in outcome seen with the introduction of immune-checkpoint inhibitors for the treatment of malignant melanoma and lung cancers has heralded a rush among biotech and pharma companies to develop new tools to activate or expand the abilities of the patient immune system to control tumor growth.

Further progress in the engineering of monoclonal antibodies lead to the development of BITE^®^ or bispecific T cell engager; the first BITE^®^ to reach the market was blinatunomab that targets CD19 and is indicated for the treatment of relapsed/refractory (r/r) adult acute lymphoblastic leukemia (ALL) since 2015. BITE© antibodies have two arms, one that binds a membrane antigen expressed at the surface of the targeted (tumor) cell such as CD19 and the other that binds T cells leading to their activation and cytotoxic effect in the close vicinity of the tumor cells [27].

**Figure 1:**
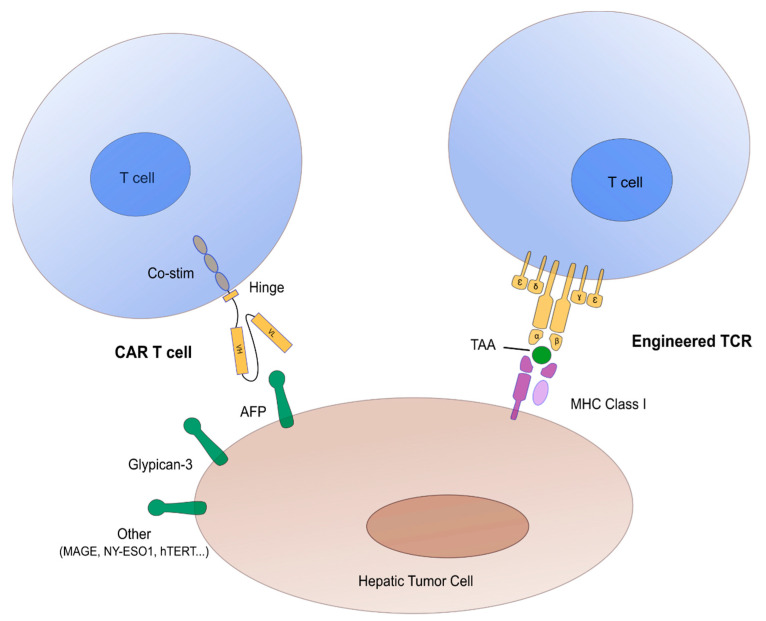
Schematic representation of T cell receptor (TCR) engineered T cells and chimeric antigen receptors (CAR) engineered T cells in hepatocellular carcinoma. Main tumor associated antigens (TAA) are represented in green. Abbreviations: AFP = alpha-fetoprotein, Co-stim = co-stimulation molecules (mainly CD28, ICOS, 41 BB, OX40), MHC Class 1 = major histocompatibility complex class I, TAA = tumor associated antigens.

Another important and more recent avenue is the development of hematopoietic cellular therapies (Figure 1) manufactured from or made of immune effector cells (IECs), the most publicized of which being CAR-T Cells [28,29]. CAR stands for ‘Chimeric Antigen Receptor’ a synthetic protein encoded by a DNA sequence that juxtaposes the extracellular domain of a single chain immunoglobulin, the intracellular domain of the Zeta chain of the T-Cell Receptor (TCR) with a hinge region and one or several domains from costimulatory molecules such as CD28 or 4.1 BB in between [30]. The extracellular domain targets a membrane antigen expressed at the surface of targeted cells such as CD19 for lymphoid malignancies (ALL, non-Hodgkin’s lymphoma, etc.) or B Cell Maturation Antigen (BCMA) expressed on malignant plasma cells in patients affected with multiple myeloma. Recognition of the target antigen is not restricted by the Major Histocompatibility Complex (MHC) molecules, allowing for wide clinical applications. Binding of the cognate ligand triggers T-cell activation and cytotoxicity through the TCR domain. The nature of the co-stimulatory domain(s) has important implications for in vivo amplification and persistence of CAR-T cells after their infusion.

Currently, three autologous CAR-T cells—all targeting CD19—have been approved by health authorities in the USA and Europe, as well as in many other countries: these include tisagenlecleucel for the treatment of relapsed/refractory ALL under the age of 25 [31] as well as for the treatment of relapsed/refractory diffuse large B-cell Lymphomas (DLBCL) [32,33], axicabtagene ciloleucel for the treatment of r/r DLBCL and primary mediastinal NHL [34] and brexucabtagene autoleucel for the treatment of r/r mantle cell NHL [35]. Two autologous CAR-T Cells targeting BCMA, idecabtagene vicleucel [36] and ciltacabtagene autoleucel [37], are likely to be soon approved by the FDA, the EMA, and Chinese health authorities for the treatment of patients affected with advanced multiple myeloma. 

Numerous developments are underway with the evaluation of novel tumor targets to treat new categories of diseases such as Hodgkin’s disease [38], myeloid malignancies [39] or solid tumors, strategies to overcome resistance, largely due to the loss of the targeted tumor antigen [40], strategies to mitigate side-effects associated with CAR-T cells administration such as the cytokine release syndrome (CRS) or immune effector cells associated neurological syndromes (ICANS) [41,42,43] improved and more complex CAR structures designed to counteract the immune suppressive environment that characterizes many tumor types, support in vivo persistence of CAR-T cells and recruit endogenous immune effectors [44]. In addition, CAR-technologies are now combined with gene editing as a substitute to retroviral or lentiviral vector transduction [45] with the use of allogeneic cells that hold the promise of off-the-shelf medicines [46] and the genetic engineering of other immune cell subsets such as natural killer (NK) cells [47], γ/δ T cells, or macrophages. The field is thus blooming with expectations.

In addition to the excitement raised by the first approved CAR-T Cells, the field of IECs is also pursuing developments with TCR transgenic T-Cells (also called engineered T-cells). In this context, recognition of the targeted tumor cells is not limited to membrane antigens but allows for the recognition of MHC-restricted peptides and may thus be more adapted to the treatment of solid tumors (Figure 1). Nevertheless, MHC restriction limits the application to subsets of patients that share the most frequent HLA types in a population of common ancestry. Editing of the endogenous TCR to be replaced by the transgenic TCR is likely to improve biological activity in the future, but similar to CAR-T cells, the issues of T-cell exhaustion in an immune suppressive tumor micro-environment and of trafficking of the genetically modified T-cells to the tumor site needs to be tackled before consistent clinical efficacy can be demonstrated and the first medicinal products in this category are approved and reach the market. Both for TCR-T cells and CAR-T cells, the choice and validation of the target antigen is of utmost importance for optimal clinical efficacy and minimization of on-target/off-tumor side-effects.

All currently available and investigational IECs represent a new category of medicinal products that require a very specific organization for the manufacturing process in the context of newly defined regulatory frameworks, as well as a very specific organization for hospitals that provide access to these treatments [48]. The complex, sophisticated, and largely manual logistics—that involves shipment of viable cells over long distances—partly explains the high price tag of these innovative gene therapy or cell therapy medicinal products. It also implies a significant turnaround time before the (autologous) therapy becomes available to the candidate patient, raising significant issues in terms of disease control during this period, with the need for bridging therapy in a proportion of patients, and patients with fast progressive tumors remaining ineligible for such approaches. Despite these uncertainties, the field is quickly moving forward and the potential for combinations with other forms of immunotherapies such as immune checkpoint inhibitors or with targeted therapies/chemotherapies fuels high expectations in the patients’ community and their families. Thorough evaluation of the safety profile and efficacy profile of these medicinal products that are mostly authorized on the basis of phase I/II registration trials, will require the collection of data over extended period of time in real-world conditions in the post-authorization era [49]. This will also help define the role of these IECs in the treatment of various categories of neoplastic diseases, in particular in comparison with other immunotherapeutic agents such as BITE© [50].

### 2.2. Targets in Hepatocellular Carcinoma Adoptive Cell Transfer

Like other immunogenic tumors, subjects undergoing hepatic resection for HCC with prominent lymphocyte infiltration are associated with reduced recurrence and better prognosis as compared with those without prominent lymphocyte infiltration [51,52]. Moreover, recurrence after liver transplantation for HCC is related to immunosuppression [53] as well as the presence of T regulatory cells (Tregs) in the infiltrate [52]. 

In the next section, we are listing the main targets used in ACT for HCC. None of these antigen are tumor-specific antigens (expressed by the tumor with minimal to no expression in normal tissue) [54]. They mainly belong to three categories of tumor antigens: (i) tumor-associated antigens: antigens whose expression is enriched but not specific to cancer cells (e.g., AFP, GPC-3); (ii) cancer–testis antigens: antigens whose expression is limited to cancer cells and reproductive tissues but not adult somatic tissue (e.g., NY-ESO-1, MAGE); (iii) viral-derived cancer antigens: antigens expressed by cancer cells derived from an oncogenic viral origin (VHB, VHC).

(1) Alpha-fetoprotein (AFP) is a 70-KDa glycoprotein found in serum of early mammalian embryos, synthesized at the site of embryonal hematopoiesis: the yolk sac [55]. After birth, the levels drop off rapidly, and by the second year only trace amounts are detectable in serum. The normal adult levels typically range between 1 and 40 ng/mL. Reappearance or high serum levels are observed in several conditions: pregnancy, hepatic disorders, and malignancies such as hepatocellular carcinomas, germ cell tumors (especially with yolk sac tumor components), breast, esophagus, cervical, pancreatic, endometrial, gastric, lung, and rectum cancers [56]. Up to 50% of HCC tumors express AFP [57]. Tumor AFP expression generally correlates with serum AFP, although this correlation is not absolute. Expression of AFP in nonmalignant liver can occur, particularly in a subset of progenitor cells and during chronic inflammation, at levels typically lower than in HCC [58]. Pre-clinical studies demonstrated the potential of AFP for cellular immunotherapies [59]. It has been reported that malignant liver cells produce AFP-L3, even when HCC is at its early stages, and especially when the tumor mass is supplied by the hepatic artery.

(2) Glypican-3 (GPC-3) is a member of the heparan sulfate proteoglycan family controlling cell division and growth regulation. GPC-3 is an antigen expressed in over 70% of HCCs but rarely in non-malignant tissues. Indeed, GPC-3 positive immunostaining can differentiate hepatocellular carcinoma (HCC) from dysplastic changes in cirrhotic livers. Recent studies demonstrated that greater GPC-3 expression in tumor cells was associated with a worse prognosis for HCC [60]. Glypican-3 antibodies are investigated as a therapeutic option for HCC, either alone or as a drug carrier [61,62,63]. Numerous pre-clinical studies support the evidence of GPC-3 targeting with adoptive cell therapies [64,65].

(3) Melanoma antigen gene family (MAGE) consists of 12 members and is expressed almost exclusively in cancer tissues in a wide variety of malignant tumors [66,67,68,69]. In RNA expression in HCC, MAGE-1 and -3 were expressed in approximately 68% of the tumors; MAGE-8 was expressed in 46%; and MAGE-2, -6, -10, -11, and -12 were expressed in approximately 30% [70,71]. Several MAGE peptides have been shown to induce a strong cytotoxic T-lymphocyte (CTL) response in patients with melanoma [72,73].

(4) New York esophageal squamous cell carcinoma 1 (NY-ESO-1) is a protein consisting of 180 amino acids. As a member of the cancer testis antigen (CTA) family, NY-ESO-1 has been shown to be expressed in spermatogonia, primary spermatocytes, oogonia, and placenta and in a variety of cancers, such as melanoma, ovarian cancer, cervical cancer, gastric cancer, and HCC. [74]. In Nakamura et al., NY-ESO-1 mRNA was detected in 18 of 41 (43.9%) hepatocellular carcinomas [75].

(5) Human telomerase reverse transcriptase (hTERT) plays a key role in conferring immortality to cancer cells through the regulation of telomere length. It has been reported that 80% to 90% of hepatocellular carcinomas (HCCs) express hTERT [76]. Additionally, peptides containing hTERT epitopes are able to induce hTERT-specific cytotoxic lymphocytes [77].

(6) NK group 2 member D ligand (NKG2DL) is a type II transmembrane-anchored C-type lectin-like protein receptor expressed on natural killer (NK) cells, CD8^+^ T cells, subsets of γδ T cells, and some autoreactive CD4^+^ T cells. The Cancer Genome Atlas and microarrays of HCC samples showed NKG2DL are generally absent on the surface of normal cells but are overexpressed on malignant cells, offering good targets for CAR-T therapy. [78]. Recently, in vitro studies reported that NKG2D CAR-T cells efficiently killed the HCC cell lines.

(7) Epithelial cell adhesion molecule (EpCAM) is a type I membrane protein of 314 amino acids (aa) of which only 26 aa are facing the cytoplasm [79,80]. EpCAM has oncogenic potential and is activated by release of its intracellular domain, which can signal into the cell nucleus by engagement of elements of the wnt pathway [81]. EpCAM was found to be frequently over-expressed in a wide variety of carcinomas, including HCC, colon, gastric, pancreas, and breast cancers [82,83].

(8) Mucin1 glycoprotein 1 (MUC1) belongs to the family of human epithelial mucins [84] Its expression on normal cells is hidden from the immune system, and its aberrant glycosylation (large number of O-glycosylated tandem repeat) on tumors creates new epitopes recognized by the immune system [85]. Pre-clinical studies in vitro and in xenograft models validated MUC1 target for CAR-T therapy [86,87].

(9) Viral antigens: viral surface proteins are not controlled by available antiviral agents and are usually maintained in HCC with integrated viral genomes [88]. In vitro and in mice [89], CAR-T cells directed against the HBV surface proteins enabled human T cells to kill HBV-infected human hepatocytes and to eliminate viral DNA. Interestingly, TCR gene-modified T cells (T cells genetically engineered with a high-affinity, HLA-A2-restricted, HCV NS3:1406-1415-reactive TCR) mediated regression of established HCV^+^ HCC in xenograft model [90,91].

## 3. Results of Adoptive Cell Transfer Trials in Hepatocellular Carcinoma

We are reporting here only human clinical trials (no pre-clinical trials). At the date of the review, only early phase studies (I/II) are available.

### 3.1. TCR Engineered T Cells

In 2015, a case report of TCR engineered T cells against viral antigens (HBsAg) described that modified T cells survived expanded and mediated a reduction in HBsAg levels without exacerbation of liver inflammation or other toxicity. However, no efficacy was observed in this patient with end-stage metastatic disease [92].

At the International Liver Congress 2020 (LBO12), Sangro et al., presented interesting data about genetically engineered affinity-enhanced autologous SPEAR T-cells (AFPc332T-cells) [93]. In this first-in-human study in HCC (NCT03132792), patients must be HLAA*02:01+ or 02:642+ and had AFP expression by immunohistochemistry (IHC) at ≥1+ in ≥20% HCC cells or serum AFP ≥ 400 ng/mL, and ≤5% IHC AFP in non-cancerous liver tissue. Four patients have been treated with ~5 billion or more transduced cells (three in Cohort 3, and one in the expansion phase): one patient with complete response, one with stable disease, and two had progressive disease. Five patients were previously treated in the first two dose cohorts with doses of 100 million and 1 billion transduced cells, respectively, and all patients had best responses of stable disease. Due to lympho-depletion (fludarabine 30 mg/m2QD for 4 days and cyclophosphamide 600 mg/m2QD for 3 days) patients experienced cytopenia (up to G4 leukopenia, lymphopenia, and neutropenia) but there were no reports of T-cell related hepatic toxicity. No DLTs were reported to date. Interestingly, the first patient in Cohort 3 had a confirmed partial response (cPR) with 100% reduction in target lesions; one non-target lesion remained at week 8. This was associated with rapid and sustained decrease in serum AFP levels from 6531 ng/mL at baseline to 14 ng/mL at week 12.

### 3.2. CAR-T Cells

In August 2020, Shi et al. published results for phase I trials with GPC3-CAR-T cells [60]. In two prospective phase I studies (NCT02395250 and NCT03146234), adult patients with advanced GPC3+ HCC (Child-Pugh A) received autologous CAR-GPC3 T-cell therapy following cyclophosphamide- and fludarabine-induced lymphodepletion. A total of 13 patients received a median of 19.9 × 10^8^ CAR-GPC3 T cells by a data cutoff date of 24 July 2019. Preliminary data of efficacy are promising with two partial responses and one patient with sustained stable disease alive after 44.2 months. The OS rates at 3 years, 1 year, and 6 months were 10.5%, 42.0%, and 50.3%, respectively. However, toxicity was a major concern as cytokine release syndrome (CRS) occurred in 9/13 patients included one G5 CRS. In this patient, 20.0 × 10^8^ cells were infused, and the next day, the patient experienced severe CRS-related hypotension, fever, pulmonary edema, and elevated plasmatic IL-6 (18,000 pg/mL). After intensive care unit transfer, the patient died from multi-organ failure at day 19. Four other patients received high-dose steroids, two of whom also received the mAb tocilizumab against IL6 receptor to manage CRS.

Unfortunately, numerous completed phase 1/2 trials have not been published yet, notably targeting MUC-1 (NCT02587689) or EpCAM (NCT03013712).

## 4. Ongoing Adoptive Cell Transfer Trials in Hepatocellular Carcinoma

The following section is resuming active clinical trials in early phase for adoptive cell therapies, either for basket studies of several tumor types including hepatocellular carcinoma, either for dedicated hepatocellular carcinoma. Of note, most of these trials include only HCC patients with a good liver function (Child Pugh A).

### 4.1. TCR Engineered T Cells

At the time of this review, five early phase trials (Table 1) are recruiting for TCR engineered (TCRe) T cells targeting AFP (NCT02719782 and NCT04368182), HBV viral antigens (NCT02719782, NCT03899415) or MAGEA1 (NCT03441100). Two trials are basket trials for solid tumors, and three trials are specific to hepatocellular carcinoma. All of these trials required a good liver function (Child A). The mains endpoints are safety (dose-limiting toxicity, adverse events, serious adverse events) and overall response rate. Four trials are conducted in unresectable hepatocellular carcinoma, and interestingly, one trial (NCT03899415) in the adjuvant setting, evaluating the safety and clinical benefit of TCR engineered T-cells therapy in patients with HBV^+^ HCC post hepatectomy or radiofrequency ablation. Results of these trials are expected after 2022.

### 4.2. CAR-T Cells

Ongoing CAR-T cells clinical trials are more frequent than TCR engineered ones (Table 2). The main target is Glypican-3 with five ongoing clinical trials. Interestingly, one of these trials (NCT03198546) investigates a CAR-T-cells targeting GPC3 and/or soluble TGFβ. The other are targeting only GPC3 using divers T cells constructions, notably with a 4-1BB Zeta chain [65].

Basket trials mostly investigate less conventional target, such as EpCAM in EpCAM positive cancer (HCC, gastro-intestinal cancer, liver cancer) (NCT03013712) or Claudin18.2, a protein implied in tight junctions and expressed in digestive tumors (stomach, gastroesophageal junction, pancreas, and liver) (NCT03302403). Baskets trials in HCC can also share several targets such as DR5, C-met, or EGFRvIII, investigated together in two CAR-T cells trials (NCT03638206, NCT03941626).

Concerning treatment characteristics, whereas intravenous infusion is the standard, one trial (NCT03993743) is investing hepatic artery infusion with four doses of CD147-CAR-T cells planned at 1-week intervals. Additionally, one trial (NCT03980288) in the cohort expansion is investigating the safety of combining CAR-T cells (CAR-GPC3) with currently available treatments for HCC, TKI, or PD-1/PD-L1 monoclonal antibody.

Most of those trials are phase 1 trials, evaluating the safety, notably the maximum tolerated dose (MTD) usually expressed in number of reinfused CAR-T cells and the number of patients with dose limiting toxicity (DLT). Secondary endpoints mostly include T cell persistence in peripheral blood, response (best response, duration of response) and rarely survival characteristics (PFS, OS). Results of these trials are expected around after 2023.

### 4.3. Immune Monitoring of ACT 

The monitoring of engineered TCR or CAR T cells is principally done by measuring the persistence by DNA copy number (copies/mg genomic DNA) [60]. Interestingly, single cell analysis techniques (notably flow cytometry) precise the composition of ACT products (proportion CD4^+^ and CD8^+^ T cell subsets) [94]. Indeed, in patients with non-Hodgkin’s lymphoma, naive and central memory CD4^+^ CAR-T cells from patients exhibit higher antitumor activity than effector memory CD4^+^ CAR-T cells when transferred to an animal model. In addition, central memory CD8^+^ CAR-T cell from patients presented higher antitumor activity than naive and effector memory CD8^+^ CAR-T cells [95].

Single cell RNA-sequencing (RNA-seq) also enables to analyze the activation state of CAR-T cells [96]. In hematologic tumors, scRNA-seq demonstrates that clones that expand after infusion mainly originate from infused clusters with higher expression of cytotoxicity and proliferation genes [97]. Moreover, single cell assays can determine their cytokines secretion and cytotoxicity [98].

In the only human phase 1 of CAR T cells published in HCC [60], CAR T-cell products were monitored by flow cytometry and were predominantly terminally differentiated effector memory T cells (CD45RA+/CCR7−; mean, 78.2%) and effector memory T cells (CD45R-CCR7-; mean, 14.1%; range, 0.8–47.4%). Characterizing precisely the quality of CAR-T infusion products and monitoring therapeutic responses is a future challenge of ACT in solid tumors.

## 5. Main Obstacles and Possible Solutions

We will describe here the main challenges for the clinical use of TCR modified and CAR-T cells therapies in HCC, including the possible strategies to overpass them [99].

### 5.1. A Specific Tumor Micro-Environment (TME)

First, the access to tumor cells (trafficking and homing) can be particularly complex for modified TCR/CAR-T cells that may face vasculature restriction or physical barrier of the stroma [100]. An adequate trafficking depends on multiple parameters, such as the matching between chemokines secreted by tumors and chemokines receptors on T cells (e.g., CXCR3 and CCR5), a good rolling on the endothelium, or an efficient extravasation and adhesion (e.g., ICAM-1 and VCAM-1) to the extracellular matrix [101]. One of the possible solutions is the intra-tumoral injection of the ACT product (NCT03993743), or the engineering of T cells able to target components of the matrix, such as αvβ6 integrin28, VEGF receptor-2, or fibroblast activation protein [102,103].

Second, in solid tumors, engineered cells must face an immunosuppressive TME composed of cellular actors (MDSC, M2 macrophages, TRegs, BRegs, DCRegs, CAF) and of immunosuppressive cytokines (IL10, TGFB). These cells produce reactive oxygen/nitrogen species, and arginase suppressing T cells functions [104]. To overpass oxidative stress, one approach is to engineer CAR-T cells coexpressing catalase (an enzyme that reduces hydrogen peroxide to water and oxygen) or HIF1α [105,106]. However, these solutions are still pre-clinical.

### 5.2. Antigen Heterogeneity

Ideally, the antigen chosen for a target in ACT needs to be highly expressed on the surface of tumor cells and not by essential healthy tissues [107]. However, exome sequencing of primary tumors and metastatic site provides evidence of intratumor heterogeneity with spatially separated heterogeneous somatic mutations and chromosomal imbalances [108]. Moreover, tumor formation involves the co-evolution of neoplastic cells together with extracellular matrix and vascular endothelial, stromal, and immune cells, which may increase their diversity [109]. This antigen heterogeneity decreases the efficacy of ACT in solid tumors. Additionally, antigen loss is often observed in solid tumor patient treated with CAR-T cells (e.g., loss if EGFRvIII in gliobastoma) [110].

A possible solution to overpass tumor heterogeneity and antigen loss is to engineer T cells targeting multiple antigens simultaneously [111]. Using an OR-gate approach, wherein either one CAR receptor can be engineered to contain two antigen binding domains (e.g., a bi-specific or tandem CAR) or two CAR receptors can be expressed on the same T cell (e.g., a bicistronic or co-transduced CAR), CAR-T cell activation occurs if either antigen is present on the cell surface [56]. A more simple approach is to enhance immunogenic cell death (chemotherapy, oncolytic viruses) to improve neoantigens release [112].

### 5.3. Limited Expansion and Persistence

Modified TCR T cells and CAR-T cells can eliminate relapsed and refractory tumors, but the durability of antitumor activity requires in vivo persistence [113]. Robust in vivo expansion and persistence of genetically modified T cells are considered critical predictors of durable clinical remissions in patients with hematologic malignancies [114]. Even in hematological trial targeting CD19; 40–60% of patients relapse owing to poor CAR-T cell persistence [115]. Very few persistence data about engineered T cells in HCC are currently available. In the published phase 1 of GPC3-CAR-T cells, the median CAR-GPC3 DNA copy number in the peripheral blood of all patients increased rapidly, reaching a peak of 360.4 copies/mg genomic DNA (range, 28.0–23,358.0 copies/mg genomic DNA) after a median period of 10.5 days (mean, 13.8 days) and lasting for a median duration of 19.5 days (mean, 34.4 days).

Technical solutions to enhance persistence were extensively reviewed elsewhere [116] and notably include: (i) the improvement of intracellular co-stimulation (e.g., CD28); (ii) the manipulation of T cells to express cytokines and their receptors (IL15–IL21: GPC3-CAR-T cells) [117]; (iii) the combination with PD(L)1 inhibitors; (iv) the modification of conditioning regimen (for example the use of conditioning with 5-Azacytidine or Fludarabine) [118].

### 5.4. Off-Target Toxicity

Toxicity is one of the most delicate points in the management of ACT in HCC. As targeted antigens can be shared be non-tumoral tissue, the risk of severe off-target toxicity is high. Concerning cytokines releasing syndrome (CRS), in the previously reported trial of CAR-T cells targeting GPC-3, CRS occurred in 9/13 patients included one G5 CRS (toxic death on day 19) [60]. Grade 5 CRS were already described in solid tumor, notably when targeting ERBB2 [119], with the hypothesis that administrated cells recognized low level of the target antigen in lung epithelial cells. Other organ-specific toxicities occur in solid tumors when using ACT, notably G4 colitis or G4 hepatitis that may be particularly dreadful in the context of HCC [120,121]. 

To deal with this major concern, genetic manipulations of T cells were developed to modify the affinity of TCR (=Low Affinity CAR-T) to spare normal tissues [122,123]. The use of affinity-tuned scFvs may empower wider use of CAR-T cells against validated targets. The insertion of suicide gene into CAR-T cells (e.g., dimerization domain fused to a caspase-9 domain) is also promising [124]. The use of effectors cells inducible by IL-12 are in evaluation in HCC [125]. Finally, recent works in HCC report the splitting of CAR in two parts (split GPC-3 CAR-T cells) using a sequence of 13 amino acid (SpyTag) in order to decrease the amount of proinflammatory cytokines released [126].

### 5.5. Clinical Applicability

A major limitation of ACT transfer in routine clinic in HCC is related with the number of selection criteria in early clinical trials. First, in TCR engineered T cells, HLA restriction lead to the loss of an important number of patients as main protocols are developed for HLA *02:01 patients (around 40% of total). Additionally, most of the trials require a hepatic function with Child Pugh class A, 7 to 9. Lastly, if the mandatory ECOG 0-1 if added, the potential frequency of patients likely to benefit from these treatments become very low. Finally, cost limitations will certainly be a barrier for health insurance system if these treatments cost the same price than in hematology (several hundred thousand dollars).

If validated in phase 3 studies, improving the cost-effectiveness and the routine clinic transfer of adoptive cell therapies in HCC will be a major challenge.

## 6. Conclusions

After years of fundamental research, adoptive cell therapy is emerging in advanced HCC. Fifteen early phase clinical trials are ongoing and two of them (GPC3-CAR-T and SPEAR AFPc332 T cells) are reporting objective responses in pre-treated advanced HCC. These first results suggest that the field is switching from an experimental proof of concept phase to an active clinical phase, with clear efficacy data. However, these trials require confirmation in a phase 3 setting. Numerous questions remain, such as the best antigen to target, the combination with ICI, the management of toxicity, the monitoring of the product and the cost of these treatments. However, it seems clear that immunotherapy will play a pivotal role in advanced HCC. Because of major advances in synthetic biology, ACT will probably become a safer and more efficient therapy that could integrate advanced HCC treatment in the next decade.

## Figures and Tables

**Table 1:** Ongoing clinical trials of TCR engineered (TCRe) T cells in hepatocellular carcinoma.

NCCT	Phase	Type	Product	Target	Organ	Pop	Sponsor	Primary Endpoint	Start Date
NCT02719782	I	TCRe	HBV-TCR T cell	HBV Ag	HCC HBV+	10	Lion Company	Safety	06/2015
NCT03132792	I	TCRe	AFPᶜ^332^ T cells	AFP	basket	45	Adaptimmune	Safety	05/2017
NCT03441100	I	TCRe	IMA202-101	MAGEA1	basket	15	Immatics	Safety	05/2019
NCT03899415	I	TCRe	HBV-TCR T cell	HBV Ag	HCC HBV+	10	Beijing Hospit.	Safety	06/2019
NCT04368182	I	TCRe	Autologous C-TCR055	AFP	HCC	5	Zhejiang Univ.	Safety	06/2020

Abbreviations: Ag: antigen; AFP: alpha-fetoprotein; HBV: hepatitis B virus; HCC: hepatocellular carcinoma; MAGE: melanoma antigen gene, TCRe: TCR engineered T cells.

**Table 2:** Ongoing clinical trials of chimeric antigen receptor (CAR) T cells in hepatocellular carcinoma.

NCCT	Phase	Type	Product	Target	Organ	Pop	Sponsor	Primary Endpoint	Start Date
NCT03302403	NA	CAR-T	CAR-CLD18	CLD18	HCC/basket	48	Kang YU	Safety	11/2017
NCT03198546	I	CAR-T	GPC3/TGFβ-CART	GPC3/TGFβ	HCC	30	Guangzhou Medical Univ	Safety	07/2017
NCT03013712	I-II	CAR-T	EpCAM-CAR-T	EpCAM	basket	60	Chengdu Medical Coll	Safety	01/2018
NCT02905188	I	CAR-T	GLYCAR	GP3C	HCC	14	Baylor Coll of Medicine	Safety	03/2019
NCT03884751	I	CAR-T	CAR-GPC3 T Cells	GPC3	HCC	15	Carsgen Therapeutics	Safety	04/2019
NCT03993743	I (IAH)	CAR-T	CD147-CART	CD147	HCC	34	Xijing Hospital	Safety	05/2019
NCT03980288	I	CAR-T	CAR-GPC3 T Cells	GPC3	HCC	36	Zhejiang Univ	Safety	07/2019
NCT03638206	I-II	CAR-T	CAR-T/TCR-T cell	DR5, C-met, EGFR vIII	basket	73	Shenzhen BinDeBio	Safety	08/2020
NCT03941626	I-II	CAR-T	CAR-T/TCR-T cells	DR5, EGFR vIII	HCC/basket	50	Shenzhen BinDeBio	Safety	09/2019
NCT04121273	I	CAR-T	NA	GPC3	HCC	20	Nanjing Univ	Safety	10/2019

AFP: alpha-fetoprotein; CLD18: claudin18; DR5: death receptor 5; HBV: hepatitis B virus; EpCAM: epithelial cell adhesion molecule; GPC3: glypican 3; HCC: hepatocellular carcinoma; MAGE: melanoma antigen gene, TCRe: TCR engineered T cells. TGFβ: transforming growth factor β.

## Data Availability

No new data were created or analyzed in this study. Data sharing is not applicable to this article.

## Abbreviations

ACT	Adoptive cell transfer
AFP	Alpha-fetoprotein
CAR	Chimeric antigen receptor
CIK	Cytokine-induced killer
CTL	Cytotoxic T lymphocyte
GPC3	Glypican-3
HBV	Hepatitis B virus
HCC	Hepatocellular carcinoma
HCV	Hepatitis C virus
IEC	Immune effector cells
IFN	Interferon
LAK	Lymphokine-activated killer
MAGE	Melanoma antigen gene
NK	Natural killer
TAA	Tumor-associated antigen
TCR	T cell receptor
TIL	Tumor-infiltrating lymphocyte

## References

[1] Brar G., Greten T.F., Graubard B.I., McNeel T.S., Petrick J.L., McGlynn K.A., Altekruse S.F. (2020). Hepatocellular Carcinoma Survival by Etiology: A SEER-Medicare Database Analysis. Hepatol. Commun..

[2] WHO (2020). Cancer Fact Sheet. Globocan.

[3] Anstee Q.M., Reeves H.L., Kotsiliti E., Govaere O., Heikenwalder M. (2019). From NASH to HCC: Current concepts and future challenges. Nat. Rev. Gastroenterol. Hepatol..

[4] Maucort-Boulch D., de Martel C., Franceschi S., Plummer M. (2018). Fraction and incidence of liver cancer attributable to hepatitis B and C viruses worldwide. Int. J. Cancer.

[5] Mazzaferro V., Regalia E., Doci R., Andreola S., Pulvirenti A., Bozzetti F., Montalto F., Ammatuna M., Morabito A., Gennari L. (1996). Liver Transplantation for the Treatment of Small Hepatocellular Carcinomas in Patients with Cirrhosis. N. Engl. J. Med..

[6] Maggs J.R.L., Suddle A.R., Aluvihare V., Heneghan M.A. (2012). Systematic review: The role of liver transplantation in the management of hepatocellular carcinoma. Aliment. Pharmacol. Ther..

[7] Vogel A., Cervantes A., Chau I., Daniele B., Llovet J.M., Meyer T., Nault J.-C., Neumann U., Ricke J., Sangro B. (2018). Hepatocellular carcinoma: ESMO Clinical Practice Guidelines for diagnosis, treatment and follow-up††FootnotesApproved by the ESMO Guidelines Committee: August 2018. Ann. Oncol..

[8] Burroughs A., Hochhauser D., Meyer T. (2004). Systemic treatment and liver transplantation for hepatocellular carcinoma: Two ends of the therapeutic spectrum. Lancet Oncol..

[9] Lai C.-L., Lok A.S.-F., Wu P.-C., Chan G.C.-B., Lin H.-J. (1988). Doxorubicin versus No Antitumor Therapy in Inoperable Hepatocellular Carcinoma. A Prospective Randomized Trial. Cancer.

[10] Llovet J.M., Ricci S., Mazzaferro V., Hilgard P., Gane E., Blanc J.-F., de Oliveira A.C., Santoro A., Raoul J.-L., Forner A. (2008). Sorafenib in advanced hepatocellular carcinoma. N. Engl. J. Med..

[11] Bruix J., Qin S., Merle P., Granito A., Huang Y.-H., Bodoky G., Pracht M., Yokosuka O., Rosmorduc O., Breder V. (2017). Regorafenib for patients with hepatocellular carcinoma who progressed on sorafenib treatment (RESORCE): A randomised, double-blind, placebo-controlled, phase 3 trial. Lancet Lond. Engl..

[12] Abou-Alfa G.K., Meyer T., Cheng A.-L., El-Khoueiry A.B., Rimassa L., Ryoo B.-Y., Cicin I., Merle P., Chen Y., Park J.-W. (2018). Cabozantinib in Patients with Advanced and Progressing Hepatocellular Carcinoma. N. Engl. J. Med..

[13] Kudo M., Finn R.S., Qin S., Han K.-H., Ikeda K., Piscaglia F., Baron A., Park J.-W., Han G., Jassem J. (2018). Lenvatinib versus sorafenib in first-line treatment of patients with unresectable hepatocellular carcinoma: A randomised phase 3 non-inferiority trial. Lancet.

[14] Zhu A.X., Kang Y.-K., Yen C.-J., Finn R.S., Galle P.R., Llovet J.M., Assenat E., Brandi G., Lim H.Y., Pracht M. (2018). REACH-2: A randomized, double-blind, placebo-controlled phase 3 study of ramucirumab versus placebo as second-line treatment in patients with advanced hepatocellular carcinoma (HCC) and elevated baseline alpha-fetoprotein (AFP) following first-line sorafenib. J. Clin. Oncol..

[15] El-Khoueiry A.B., Sangro B., Yau T., Crocenzi T.S., Kudo M., Hsu C., Kim T.-Y., Choo S.-P., Trojan J., Welling T.H. (2017). Nivolumab in patients with advanced hepatocellular carcinoma (CheckMate 040): An open-label, non-comparative, phase 1/2 dose escalation and expansion trial. Lancet.

[16] Finn R.S., Ryoo B.-Y., Merle P., Kudo M., Bouattour M., Lim H.Y., Breder V., Edeline J., Chao Y., Ogasawara S. (2020). Pembrolizumab As Second-Line Therapy in Patients with Advanced Hepatocellular Carcinoma in KEYNOTE-240: A Randomized, Double-Blind, Phase III Trial. J. Clin. Oncol. Off. J. Am. Soc. Clin. Oncol..

[17] Finn R.S., Qin S., Ikeda M., Galle P.R., Ducreux M., Kim T.-Y., Kudo M., Breder V., Merle P., Kaseb A.O. (2020). Atezolizumab plus Bevacizumab in Unresectable Hepatocellular Carcinoma. N. Engl. J. Med..

[18] Greten T.F., Sangro B. (2018). Targets for immunotherapy of liver cancer. J. Hepatol..

[19] Iñarrairaegui M., Melero I., Sangro B. (2018). Immunotherapy of Hepatocellular Carcinoma: Facts and Hopes. Clin. Cancer Res..

[20] Kubes P., Jenne C. (2018). Immune Responses in the Liver. Annu. Rev. Immunol..

[21] Rochigneux P., Nault J.-C., Mallet F., Chretien A.-S., Barget N., Garcia A.J., Pozo L.D., Bourcier V., Blaise L., Grando-Lemaire V. (2019). Dynamic of systemic immunity and its impact on tumour recurrence after radiofrequency ablation of hepatocellular carcinoma. OncoImmunology.

[22] Ringelhan M., Pfister D., O’Connor T., Pikarsky E., Heikenwalder M. (2018). The Immunology of Hepatocellular Carcinoma. Nat. Immunol..

[23] Calmels B., Mfarrej B., Chabannon C. (2018). From clinical proof-of-concept to commercialization of CAR-T cells. Drug Discov. Today.

[24] Brown C.E., Alizadeh D., Starr R., Weng L., Wagner J.R., Naranjo A., Ostberg J.R., Blanchard M.S., Kilpatrick J., Simpson J. (2016). Regression of Glioblastoma after Chimeric Antigen Receptor T-Cell Therapy. N. Engl. J. Med..

[25] Louis C.U., Savoldo B., Dotti G., Pule M., Yvon E., Myers G.D., Rossig C., Russell H.V., Diouf O., Liu E. (2011). Antitumour activity and long-term fate of chimeric antigen receptor–positive T cells in patients with neuroblastoma. Blood.

[26] Comoli P., Chabannon C., Koehl U., Lanza F., Urbano-Ispizua A., Hudecek M., Ruggeri A., Secondino S., Bonini C., Pedrazzoli P. (2019). Development of adaptive immune effector therapies in solid tumors. Ann. Oncol..

[27] Goebeler M.-E., Bargou R.C. (2020). T cell-engaging therapies—BiTEs and beyond. Nat. Rev. Clin. Oncol..

[28] Sadelain M., Rivière I., Riddell S. (2017). Therapeutic T cell engineering. Nature.

[29] June C.H., O’Connor R.S., Kawalekar O.U., Ghassemi S., Milone M.C. (2018). CAR-T cell immunotherapy for human cancer. Science.

[30] Lindner S.E., Johnson S.M., Brown C.E., Wang L.D. (2020). Chimeric antigen receptor signaling: Functional consequences and design implications. Sci. Adv..

[31] Maude S.L., Laetsch T.W., Buechner J., Rives S., Boyer M., Bittencourt H., Bader P., Verneris M.R., Stefanski H.E., Myers G.D. (2018). Tisagenlecleucel in Children and Young Adults with B-Cell Lymphoblastic Leukemia. N. Engl. J. Med..

[32] Schuster S.J., Svoboda J., Chong E.A., Nasta S.D., Mato A.R., Anak Ö., Brogdon J.L., Pruteanu-Malinici I., Bhoj V., Landsburg D. (2017). Chimeric Antigen Receptor T Cells in Refractory B-Cell Lymphomas. N. Engl. J. Med..

[33] Schuster S.J., Bishop M.R., Tam C.S., Waller E.K., Borchmann P., McGuirk J.P., Jäger U., Jaglowski S., Andreadis C., Westin J.R. (2019). Tisagenlecleucel in Adult Relapsed or Refractory Diffuse Large B-Cell Lymphoma. N. Engl. J. Med..

[34] Neelapu S.S., Locke F.L., Bartlett N.L., Lekakis L.J., Miklos D.B., Jacobson C.A., Braunschweig I., Oluwole O.O., Siddiqi T., Lin Y. (2017). Axicabtagene Ciloleucel CAR-T-Cell Therapy in Refractory Large B-Cell Lymphoma. N. Engl. J. Med..

[35] Wang M., Munoz J., Goy A., Locke F.L., Jacobson C.A., Hill B.T., Timmerman J.M., Holmes H., Jaglowski S., Flinn I.W. (2020). KTE-X19 CAR-T-Cell Therapy in Relapsed or Refractory Mantle-Cell Lymphoma. N. Engl. J. Med..

[36] Raje N., Berdeja J., Lin Y., Siegel D., Jagannath S., Madduri D., Liedtke M., Rosenblatt J., Maus M.V., Turka A. (2019). Anti-BCMA CAR-T-Cell Therapy bb2121 in Relapsed or Refractory Multiple Myeloma. N. Engl. J. Med..

[37] Berdeja J.G., Madduri D., Usmani S.Z., Singh I., Zudaire E., Yeh T.-M., Allred A.J., Olyslager Y., Banerjee A., Goldberg J.D. (2020). Update of CARTITUDE-1: A phase Ib/II study of JNJ-4528, a B-cell maturation antigen (BCMA)-directed CAR-T-cell therapy, in relapsed/refractory multiple myeloma. J. Clin. Oncol..

[38] Ramos C.A., Grover N.S., Beaven A.W., Lulla P.D., Wu M.-F., Ivanova A., Wang T., Shea T.C., Rooney C.M., Dittus C. (2020). Anti-CD30 CAR-T Cell Therapy in Relapsed and Refractory Hodgkin Lymphoma. J. Clin. Oncol..

[39] Bôle-Richard E., Fredon M., Biichlé S., Anna F., Certoux J.-M., Renosi F., Tsé F., Molimard C., Valmary-Degano S., Jenvrin A. (2020). CD28/4-1BB CD123 CAR-T cells in blastic plasmacytoid dendritic cell neoplasm. Leukemia.

[40] Sotillo E., Barrett D.M., Black K.L., Bagashev A., Oldridge D., Wu G., Sussman R., Lanauze C., Ruella M., Gazzara M.R. (2015). Convergence of Acquired Mutations and Alternative Splicing of CD19 Enables Resistance to CART-19 Immunotherapy. Cancer Discov..

[41] Lee D.W., Santomasso B.D., Locke F.L., Ghobadi A., Turtle C.J., Brudno J.N., Maus M.V., Park J.H., Mead E., Pavletic S. (2019). ASTCT Consensus Grading for Cytokine Release Syndrome and Neurologic Toxicity Associated with Immune Effector Cells. Biol. Blood Marrow Transplant..

[42] Yáñez L., Alarcón A., Sánchez-Escamilla M., Perales M.-A. (2020). How I treat adverse effects of CAR-T cell therapy. ESMO Open.

[43] Mestermann K., Giavridis T., Weber J., Rydzek J., Frenz S., Nerreter T., Mades A., Sadelain M., Einsele H., Hudecek M. (2019). The tyrosine kinase inhibitor dasatinib acts as a pharmacologic on/off switch for CAR-T cells. Sci. Transl. Med..

[44] Holzinger A., Abken H. (2019). CAR-T Cells: A Snapshot on the Growing Options to Design a CAR. HemaSphere.

[45] Stadtmauer E.A., Fraietta J.A., Davis M.M., Cohen A.D., Weber K.L., Lancaster E., Mangan P.A., Kulikovskaya I., Gupta M., Chen F. (2020). CRISPR-engineered T cells in patients with refractory cancer. Science.

[46] Depil S., Duchateau P., Grupp S.A., Mufti G., Poirot L. (2020). Off-the-shelf’ allogeneic CAR-T cells: Development and challenges. Nat. Rev. Drug Discov..

[47] Liu E., Marin D., Banerjee P., Macapinlac H.A., Thompson P., Basar R., Nassif Kerbauy L., Overman B., Thall P., Kaplan M. (2020). Use of CAR-Transduced Natural Killer Cells in CD19-Positive Lymphoid Tumors. N. Engl. J. Med..

[48] Yakoub-Agha I., Chabannon C., Bader P., Basak G.W., Bonig H., Ciceri F., Corbacioglu S., Duarte R.F., Einsele H., Hudecek M. (2020). Management of adults and children undergoing chimeric antigen receptor T-cell therapy: Best practice recommendations of the European Society for Blood and Marrow Transplantation (EBMT) and the Joint Accreditation Committee of ISCT and EBMT (JACIE). Haematologica.

[49] Pasquini M.C., Hu Z.-H., Curran K., Laetsch T., Locke F., Rouce R., Pulsipher M.A., Phillips C.L., Keating A., Frigault M.J. (2020). Real-world evidence of tisagenlecleucel for pediatric acute lymphoblastic leukemia and non-Hodgkin lymphoma. Blood Adv..

[50] Batlevi C.L., Matsuki E., Brentjens R.J., Younes A. (2016). Novel immunotherapies in lymphoid malignancies. Nat. Rev. Clin. Oncol..

[51] Wada Y., Nakashima O., Kutami R., Yamamoto O., Kojiro M. (1998). Clinicopathological study on hepatocellular carcinoma with lymphocytic infiltration. Hepatology.

[52] Unitt E., Marshall A., Gelson W., Rushbrook S.M., Davies S., Vowler S.L., Morris L.S., Coleman N., Alexander G.J.M. (2006). Tumour lymphocytic infiltrate and recurrence of hepatocellular carcinoma following liver transplantation. J. Hepatol..

[53] Vivarelli M., Bellusci R., Cucchetti A., Cavrini G., De Ruvo N., Aden A.A., La Barba G., Brillanti S., Cavallari A. (2002). Low recurrence rate of hepatocellular carcinoma after liver transplantation: Better patient selection or lower immunosuppression?. Transplantation.

[54] Smith C.C., Selitsky S.R., Chai S., Armistead P.M., Vincent B.G., Serody J.S. (2019). Alternative Tumour-Specific Antigens. Nat. Rev. Cancer.

[55] Bergstrand C.G., Csar B. Demonstration of a New Protein Fraction in Serum from the Human Fetus: Scandinavian Journal of Clinical and Laboratory Investigation: Vol 8, No 2. https://www.tandfonline.com/doi/abs/10.3109/00365515609049266.

[56] He Y., Lu H., Zhang L., Zhang L. (2019). Chapter Ten—Serum AFP levels in patients suffering from 47 different types of cancers and noncancer diseases. Progress in Molecular Biology and Translational Science.

[57] Sideras K., Bots S.J., Biermann K., Sprengers D., Polak W.G., IJzermans J.N.M., de Man R.A., Pan Q., Sleijfer S., Bruno M.J. (2015). Tumour antigen expression in hepatocellular carcinoma in a low-endemic western area. Br. J. Cancer.

[58] Docta R.Y., Ferronha T., Sanderson J.P., Weissensteiner T., Pope G.R., Bennett A.D., Pumphrey N.J., Ferjentsik Z., Quinn L.L., Wiedermann G.E. (2019). Tuning T-Cell Receptor Affinity to Optimize Clinical Risk-Benefit When Targeting Alpha-Fetoprotein–Positive Liver Cancer. Hepatology.

[59] Zhu W., Peng Y., Wang L., Hong Y., Jiang X., Li Q., Liu H., Huang L., Wu J., Celis E. (2018). Identification of α-fetoprotein-specific T-cell receptors for hepatocellular carcinoma immunotherapy. Hepatology.

[60] Shi D., Shi Y., Kaseb A.O., Qi X., Zhang Y., Chi J., Lu Q., Gao H., Jiang H., Wang H. (2020). Chimeric Antigen Receptor-Glypican-3 T-Cell Therapy for Advanced Hepatocellular Carcinoma: Results of Phase I Trials. Clin. Cancer Res..

[61] Zhu A.X., Gold P.J., El-Khoueiry A.B., Abrams T.A., Morikawa H., Ohishi N., Ohtomo T., Philip P.A. (2013). First-in-Man Phase I Study of GC33, a Novel Recombinant Humanized Antibody Against Glypican-3, in Patients with Advanced Hepatocellular Carcinoma. Clin. Cancer Res..

[62] Hanaoka H., Nagaya T., Sato K., Nakamura Y., Watanabe R., Harada T., Gao W., Feng M., Phung Y., Kim I. (2015). Glypican-3 targeted human heavy chain antibody as a drug carrier for hepatocellular carcinoma therapy. Mol. Pharm..

[63] Shimizu Y., Suzuki T., Yoshikawa T., Endo I., Nakatsura T. (2019). Next-Generation Cancer Immunotherapy Targeting Glypican-3. Front. Oncol..

[64] Jiang Z., Jiang X., Chen S., Lai Y., Wei X., Li B., Lin S., Wang S., Wu Q., Liang Q. (2017). Anti-GPC3-CAR-T Cells Suppress the Growth of Tumour Cells in Patient-Derived Xenografts of Hepatocellular Carcinoma. Front. Immunol..

[65] Li W., Guo L., Rathi P., Marinova E., Gao X., Wu M.-F., Liu H., Dotti G., Gottschalk S., Metelitsa L.S. (2016). Redirecting T Cells to Glypican-3 with 4-1BB Zeta Chimeric Antigen Receptors Results in Th1 Polarization and Potent Antitumour Activity. Hum. Gene Ther..

[66] Raghavendra A., Croft P.K., Vargas A.C., Smart C.E., Simpson P.T., Saunus J.M., Lakhani S.R. (2018). Expression of MAGE-A and NY-ESO-1 cancer/testis antigens is enriched in triple-negative invasive breast cancers. Histopathology.

[67] Kakimoto T., Matsumine A., Kageyama S., Asanuma K., Matsubara T., Nakamura T., Iino T., Ikeda H., Shiku H., Sudo A. (2019). Immunohistochemical expression and clinicopathological assessment of the cancer testis antigens NY-ESO-1 and MAGE-A4 in high-grade soft-tissue sarcoma. Oncol. Lett..

[68] Wei Y., Wang Y., Gong J., Rao L., Wu Z., Nie T., Shi D., Zhang L. (2018). High expression of MAGE-A9 contributes to stemness and malignancy of human hepatocellular carcinoma. Int. J. Oncol..

[69] Li R., Gong J., Xiao C., Zhu S., Hu Z., Liang J., Li X., Yan X., Zhang X., Li D. (2020). A comprehensive analysis of the MAGE family as prognostic and diagnostic markers for hepatocellular carcinoma. Genomics.

[70] Tahara K., Mori M., Sadanaga N., Sakamoto Y., Kitano S., Makuuchi M. (1999). Expression of the MAGE gene family in human hepatocellular carcinoma. Cancer.

[71] Kobayashi Y., Higashi T., Nouso K., Nakatsukasa H., Ishizaki M., Kaneyoshi T., Toshikuni N., Kariyama K., Nakayama E., Tsuji T. (2000). Expression of MAGE, GAGE and BAGE genes in human liver diseases: Utility as molecular markers for hepatocellular carcinoma. J. Hepatol..

[72] Marchand M., van Baren N., Weynants P., Brichard V., Dréno B., Tessier M.-H., Rankin E., Parmiani G., Arienti F., Humblet Y. (1999). Tumour regressions observed in patients with metastatic melanoma treated with an antigenic peptide encoded by gene MAGE-3 and presented by HLA-A1. Int. J. Cancer.

[73] Schooten E., Di Maggio A., van Bergen en Henegouwen P.M.P., Kijanka M.M. (2018). MAGE-A antigens as targets for cancer immunotherapy. Cancer Treat. Rev..

[74] Wang H., Chen D., Wang R., Quan W., Xia D., Mei J., Xu J., Liu C. (2019). NY-ESO-1 expression in solid tumors predicts prognosis: A systematic review and meta-analysis. Medicine.

[75] Nakamura S., Nouso K., Noguchi Y., Higashi T., Ono T., Jungbluth A., Chen Y.-T., Old L.J., Nakayama E., Shiratori Y. (2006). Expression and immunogenicity of NY-ESO-1 in hepatocellular carcinoma. J. Gastroenterol. Hepatol..

[76] Miura N., Maeda Y., Kanbe T., Yazama H., Takeda Y., Sato R., Tsukamoto T., Sato E., Marumoto A., Harada T. (2005). Serum Human Telomerase Reverse Transcriptase Messenger RNA as a Novel Tumour Marker for Hepatocellular Carcinoma. Clin. Cancer Res..

[77] Mizukoshi E., Nakamoto Y., Marukawa Y., Arai K., Yamashita T., Tsuji H., Kuzushima K., Takiguchi M., Kaneko S. (2006). Cytotoxic T cell responses to human telomerase reverse transcriptase in patients with hepatocellular carcinoma. Hepatology.

[78] Sun B., Yang D., Dai H., Liu X., Jia R., Cui X., Li W., Cai C., Xu J., Zhao X. (2019). Eradication of Hepatocellular Carcinoma by NKG2D-Based CAR-T Cells. Cancer Immunol. Res..

[79] Baeuerle P.A., Gires O. (2007). EpCAM (CD326) finding its role in cancer. Br. J. Cancer.

[80] Marhaba R., Klingbeil P., Nuebel T., Nazarenko I., Buechler M.W., Zoeller M. (2008). CD44 and EpCAM: Cancer-initiating cell markers. Curr. Mol. Med..

[81] Munz M., Baeuerle P.A., Gires O. (2009). The Emerging Role of EpCAM in Cancer and Stem Cell Signaling. Cancer Res..

[82] Yamashita T., Forgues M., Wang W., Kim J.W., Ye Q., Jia H., Budhu A., Zanetti K.A., Chen Y., Qin L.-X. (2008). EpCAM and α-Fetoprotein Expression Defines Novel Prognostic Subtypes of Hepatocellular Carcinoma. Cancer Res..

[83] Yang Y., McCloskey J.E., Yang H., Puc J., Gallegos A.A.G., Vedvyas Y., Min I.M., von Hofe E., Jin M.M. (2020). Abstract 6598: Eradication of EpCAM expressing solid tumors by low-affinity CAR-T cells. Cancer Res..

[84] Kotera Y., Fontenot J.D., Pecher G., Metzgar R.S., Finn O.J. (1994). Humoral Immunity against a Tandem Repeat Epitope of Human Mucin MUC-1 in Sera from Breast, Pancreatic, and Colon Cancer Patients. Cancer Res..

[85] Finn O.J., Jerome K.R., Henderson R.A., Pecher G., Domenech N., Magarian-Blander J., Barratt-Boyes S.M. (1995). MUC-1 epithelial tumour mucin-based immunity and cancer vaccines. Immunol. Rev..

[86] Zhou R., Yazdanifar M., Roy L.D., Whilding L.M., Gavrill A., Maher J., Mukherjee P. (2019). CAR-T Cells Targeting the Tumour MUC1 Glycoprotein Reduce Triple-Negative Breast Cancer Growth. Front. Immunol..

[87] Mei Z., Zhang K., Lam A.K.-Y., Huang J., Qiu F., Qiao B., Zhang Y. (2020). MUC1 as a target for CAR-T therapy in head and neck squamous cell carinoma. Cancer Med..

[88] Bohne F., Chmielewski M., Ebert G., Wiegmann K., Kürschner T., Schulze A., Urban S., Krönke M., Abken H., Protzer U. (2008). T Cells Redirected Against Hepatitis B Virus Surface Proteins Eliminate Infected Hepatocytes. Gastroenterology.

[89] Krebs K., Böttinger N., Huang L., Chmielewski M., Arzberger S., Gasteiger G., Jäger C., Schmitt E., Bohne F., Aichler M. (2013). T Cells Expressing a Chimeric Antigen Receptor That Binds Hepatitis B Virus Envelope Proteins Control Virus Replication in Mice. Gastroenterology.

[90] Koh S., Shimasaki N., Suwanarusk R., Ho Z.Z., Chia A., Banu N., Howland S.W., Ong A.S.M., Gehring A.J., Stauss H. (2013). A practical approach to immunotherapy of hepatocellular carcinoma using T cells redirected against hepatitis B virus. Mol. Ther. Nucleic Acids.

[91] Spear T.T., Callender G.G., Roszkowski J.J., Moxley K.M., Simms P.E., Foley K.C., Murray D.C., Scurti G.M., Li M., Thomas J.T. (2016). TCR gene-modified T cells can efficiently treat established hepatitis C-associated hepatocellular carcinoma tumors. Cancer Immunol. Immunother..

[92] Qasim W., Brunetto M., Gehring A.J., Xue S.-A., Schurich A., Khakpoor A., Zhan H., Ciccorossi P., Gilmour K., Cavallone D. (2015). Immunotherapy of HCC metastases with autologous T cell receptor redirected T cells, targeting HBsAg in a liver transplant patient. J. Hepatol..

[93] Sangro B. (2020). Contents. J. Hepatol..

[94] De Azevedo J.T.C., Mizukami A., Moço P.D., Malmegrim K.C.R., Swiech K., Malmegrim K.C.R., Picanço-Castro V. (2020). Immunophenotypic Analysis of CAR-T Cells. Chimeric Antigen Receptor T Cells: Development and Production.

[95] Sommermeyer D., Hudecek M., Kosasih P.L., Gogishvili T., Maloney D.G., Turtle C.J., Riddell S.R. (2016). Chimeric Antigen Receptor-Modified T Cells Derived from Defined CD8 + and CD4 + Subsets Confer Superior Antitumor Reactivity in Vivo. Leukemia.

[96] Li X., Guo X., Zhu Y., Wei G., Zhang Y., Li X., Xu H., Cui J., Wu W., He J. (2020). Single-Cell Transcriptomic Analysis Reveals BCMA CAR-T Cell Dynamics in a Patient with Refractory Primary Plasma Cell Leukemia. Mol. Ther..

[97] Sheih A., Voillet V., Hanafi L.-A., DeBerg H.A., Yajima M., Hawkins R., Gersuk V., Riddell S.R., Maloney D.G., Wohlfahrt M.E. (2020). Clonal Kinetics and Single-Cell Transcriptional Profiling of CAR-T Cells in Patients Undergoing CD19 CAR-T Immunotherapy. Nat. Commun..

[98] Xhangolli I., Dura B., Lee G., Kim D., Xiao Y., Fan R. (2019). Single-Cell Analysis of CAR-T Cell Activation Reveals A Mixed TH1/TH2 Response Independent of Differentiation. Genom. Proteom. Bioinform..

[99] Ramakrishna S., Barsan V., Mackall C. (2020). Prospects and challenges for use of CAR-T cell therapies in solid tumors. Expert Opin. Biol. Ther..

[100] D’Aloia M.M., Zizzari I.G., Sacchetti B., Pierelli L., Alimandi M. (2018). CAR-T cells: The long and winding road to solid tumors. Cell Death Dis..

[101] Newick K., O’Brien S., Moon E., Albelda S.M. (2017). CAR-T Cell Therapy for Solid Tumors. Annu. Rev. Med..

[102] Whilding L.M., Vallath S., Maher J. (2016). The integrin αvβ6: A novel target for CAR-T-cell immunotherapy?. Biochem. Soc. Trans..

[103] Wang L.-C.S., Lo A., Scholler J., Sun J., Majumdar R.S., Kapoor V., Antzis M., Cotner C.E., Johnson L.A., Durham A.C. (2014). Targeting Fibroblast Activation Protein in Tumour Stroma with Chimeric Antigen Receptor T Cells Can Inhibit Tumour Growth and Augment Host Immunity without Severe Toxicity. Cancer Immunol. Res..

[104] Martinez M., Moon E.K. (2019). CAR-T Cells for Solid Tumors: New Strategies for Finding, Infiltrating, and Surviving in the Tumour Microenvironment. Front. Immunol..

[105] Ligtenberg M.A., Mougiakakos D., Mukhopadhyay M., Witt K., Lladser A., Chmielewski M., Riet T., Abken H., Kiessling R. (2016). Coexpressed Catalase Protects Chimeric Antigen Receptor–Redirected T Cells as well as Bystander Cells from Oxidative Stress–Induced Loss of Antitumour Activity. J. Immunol..

[106] Juillerat A., Marechal A., Filhol J.M., Valogne Y., Valton J., Duclert A., Duchateau P., Poirot L. (2017). An oxygen sensitive self-decision making engineered CAR-T-cell. Sci. Rep..

[107] Newick K., Moon E., Albelda S.M. (2016). Chimeric antigen receptor T-cell therapy for solid tumors. Mol. Ther. Oncolytics.

[108] Gerlinger M., Rowan A.J., Horswell S., Larkin J., Endesfelder D., Gronroos E., Martinez P., Matthews N., Stewart A., Tarpey P. (2012). Intratumour heterogeneity and branched evolution revealed by multiregion sequencing. N. Engl. J. Med..

[109] Junttila M.R., de Sauvage F.J. (2013). Influence of tumour micro-environment heterogeneity on therapeutic response. Nature.

[110] O’Rourke D.M., Nasrallah M.P., Desai A., Melenhorst J.J., Mansfield K., Morrissette J.J.D., Martinez-Lage M., Brem S., Maloney E., Shen A. (2017). A single dose of peripherally infused EGFRvIII-directed CAR-T cells mediates antigen loss and induces adaptive resistance in patients with recurrent glioblastoma. Sci. Transl. Med..

[111] Grada Z., Hegde M., Byrd T., Shaffer D.R., Ghazi A., Brawley V.S., Corder A., Schönfeld K., Koch J., Dotti G. (2013). TanCAR: A Novel Bispecific Chimeric Antigen Receptor for Cancer Immunotherapy. Mol. Ther. Nucleic Acids.

[112] Ajina A., Maher J. (2017). Prospects for combined use of oncolytic viruses and CAR-T-cells. J. Immunother. Cancer.

[113] Guedan S., Madar A., Casado-Medrano V., Shaw C., Wing A., Liu F., Young R.M., June C.H., Posey A.D. (2020). Single residue in CD28-costimulated CAR-T cells limits long-term persistence and antitumour durability. J. Clin. Investig..

[114] Guedan S., Posey A.D., Shaw C., Wing A., Da T., Patel P.R., McGettigan S.E., Casado-Medrano V., Kawalekar O.U., Uribe-Herranz M. (2018). Enhancing CAR-T cell persistence through ICOS and 4-1BB costimulation. JCI Insight.

[115] Ghorashian S., Kramer A.M., Onuoha S., Wright G., Bartram J., Richardson R., Albon S.J., Casanovas-Company J., Castro F., Popova B. (2019). Enhanced CAR-T cell expansion and prolonged persistence in pediatric patients with ALL treated with a low-affinity CD19 CAR. Nat. Med..

[116] Jafarzadeh L., Masoumi E., Fallah-Mehrjardi K., Mirzaei H.R., Hadjati J. (2020). Prolonged Persistence of Chimeric Antigen Receptor (CAR) T Cell in Adoptive Cancer Immunotherapy: Challenges and Ways Forward. Front. Immunol..

[117] Batra S.A., Rathi P., Guo L., Courtney A.N., Fleurence J., Balzeau J., Shaik R.S., Nguyen T.P., Wu M.-F., Bulsara S. (2020). Glypican-3–Specific CAR-T Cells Coexpressing IL15 and IL21 Have Superior Expansion and Antitumour Activity against Hepatocellular Carcinoma. Cancer Immunol. Res..

[118] Dolnikov A., Yang S., Shen S., Xu N., Chaudhry K., Nordon R., O’Brien T. (2016). Prolonging CART Cell Persistence Using Conditioning with 5-Azacytidine. Cytotherapy.

[119] Morgan R.A., Yang J.C., Kitano M., Dudley M.E., Laurencot C.M., Rosenberg S.A. (2010). Case Report of a Serious Adverse Event Following the Administration of T Cells Transduced with a Chimeric Antigen Receptor Recognizing ERBB2. Mol. Ther..

[120] Parkhurst M.R., Yang J.C., Langan R.C., Dudley M.E., Nathan D.-A.N., Feldman S.A., Davis J.L., Morgan R.A., Merino M.J., Sherry R.M. (2011). T Cells Targeting Carcinoembryonic Antigen Can Mediate Regression of Metastatic Colorectal Cancer but Induce Severe Transient Colitis. Mol. Ther..

[121] Lamers C.H., Sleijfer S., van Steenbergen S., van Elzakker P., van Krimpen B., Groot C., Vulto A., den Bakker M., Oosterwijk E., Debets R. (2013). Treatment of Metastatic Renal Cell Carcinoma with CAIX CAR-engineered T cells: Clinical Evaluation and Management of On-target Toxicity. Mol. Ther..

[122] Liu X., Jiang S., Fang C., Yang S., Olalere D., Pequignot E.C., Cogdill A.P., Li N., Ramones M., Granda B. (2015). Affinity-tuned ErbB2 or EGFR chimeric antigen receptor T cells exhibit an increased therapeutic index against tumors in mice. Cancer Res..

[123] Park S., Shevlin E., Vedvyas Y., Zaman M., Park S., Hsu Y.-M.S., Min I.M., Jin M.M. (2017). Micromolar affinity CAR-T cells to ICAM-1 achieves rapid tumour elimination while avoiding systemic toxicity. Sci. Rep..

[124] Amatya C., Pegues M.A., Lam N., Vanasse D., Geldres C., Choi S., Hewitt S.M., Feldman S.A., Kochenderfer J.N. (2020). Development of CAR-T Cells Expressing a Suicide Gene Plus a Chimeric Antigen Receptor Targeting Signaling Lymphocytic-Activation Molecule F7. Mol. Ther..

[125] Liu Y., Di S., Shi B., Zhang H., Wang Y., Wu X., Luo H., Wang H., Li Z., Jiang H. (2019). Armored Inducible Expression of IL-12 Enhances Antitumour Activity of Glypican-3–Targeted Chimeric Antigen Receptor–Engineered T Cells in Hepatocellular Carcinoma. J. Immunol..

[126] Liu X., Wen J., Yi H., Hou X., Yin Y., Ye G., Wu X., Jiang X. (2020). Split chimeric antigen receptor-modified T cells targeting glypican-3 suppress hepatocellular carcinoma growth with reduced cytokine release. Ther. Adv. Med. Oncol..

